# A Very Romantic History

**Published:** 1882-04

**Authors:** 


### A VERY ROMANTIC HISTORY.

The story of the Countess Benedetti, wife of the French minister to Prussia, is worth recording. She was once a Greek slave, landed at Alexandria by Jocosi, the celebrated merchant of Constantinople. She had been educated for sale, and was consequently full of accomplishments. One of the wealthiest Arab merchants in Alexandria purchased the girl to wait upon his wife, to whom he was much attached. The Greek girl, lively and amusing, diverted the *ennui* of the household, and soon became the ruling spirit.

In the course of time the wife died, and the aged husband, regretting that he could not marry her, adopted her as his child, and the heir to his enormous fortune.

At his death, the former slave inherited his wealth, and Benedetti, at that time a young attaché belonging to the French Consulate at Alexandria, happened to present himself to the heiress, won her affections, and they were married. The old merchant's money enabled Benedetti to cut his way to a conspicuous position in diplomacy, and his wife, lovely and accomplished, reigned for a long time over the world of fashion in Paris.—*Philadelphia Saturday Night.*

